# Fibroblast-derived NEU1 as a therapeutic target for improving cortical bone integrity and fracture healing

**DOI:** 10.7150/thno.119200

**Published:** 2026-01-01

**Authors:** Yutong Wu, Hongbo Ai, Chengmin Zhang, Ying Qu, Jiulin Tan, Nathachit Limjunyawong, Jianzhong Xu, Shiwu Dong, Fei Luo, Ce Dou

**Affiliations:** 1 Department of Orthopedics, Southwest Hospital, Third Military Medical University (Army Medical University), Chongqing 400038, China.; 2 Center of Research Excellence in Allergy & Immunology, Research Department, Faculty of Medicine Siriraj Hospital, Mahidol University, Bangkok 10700, Thailand.

**Keywords:** bone homeostasis, osteoclast, fracture healing, fibroblast, sialidase

## Abstract

**Rationale:** Bone fractures, particularly in aging populations, present significant clinical challenges due to prolonged healing times and increased risk of complications. A deeper understanding of the molecular mechanisms regulating bone metabolism and repair is essential for developing novel therapeutic strategies.

**Methods:** Through performing immunohistochemical staining and observation on the hind limbs of mice, we evaluated the differences in the spatial distribution of osteoclasts and sialic acid-enriched regions, and further investigated the correlation between osteoclast activation and sialic acid levels in the bone microenvironment. Additionally, single-cell sequencing was conducted to infer the main cell subsets involved in the modification of sialic acid levels in the periosteum. Meanwhile, *in vitro* and *in vivo* models were employed to specifically interfere with neuraminidase 1 (NEU1) activity, so as to verify the effectiveness of targeted regulation of NEU1 in modulating local osteoclast activation, maintaining cortical bone homeostasis, and regulating fracture healing rate.

**Results:** Through single-cell RNA sequencing of human periosteum, we discovered that NEU1 desialylates α2,3-linked sialic acid residues on osteoclasts, disrupting cell-cell recognition and maintaining osteoclasts in a mononuclear state. This mechanism contributes to the low metabolic activity and structural integrity of cortical bone while enhancing bone anabolic effects. Importantly, targeted inhibition of NEU1 in mouse models accelerated fracture healing, reducing healing time by up to 30% compared to controls and significantly improving bone quality and mechanical strength.

**Conclusions:** Here, we identify a critical role for periosteal fibroblast-derived NEU1 in modulating osteoclast activity and bone homeostasis. These findings suggest that NEU1 is a promising therapeutic target for enhancing bone regeneration and treating metabolic bone diseases. Our study lays the groundwork for the development of NEU1-targeted therapies that could transform clinical practice by promoting faster and more effective bone healing.

## Introduction

Bone fractures, particularly in aging populations, pose a significant health burden due to prolonged healing times and increased risk of complications. Understanding the cellular and molecular mechanisms that govern bone regeneration and metabolism is crucial for developing targeted therapies to enhance bone repair. Cortical and trabecular bones exhibit distinct metabolic profiles, characterized by differences in density, porosity, and metabolic activity. Cortical bone, known for its high density and low porosity, exhibits relatively low metabolic activity. In contrast, trabecular bone has a honeycomb-like architecture with thin bony struts, creating a large surface area exposed to bone marrow and blood flow, which results in a high turnover rate and increased metabolic activity 1. Periosteum is a double-layered membrane that covers the outer surface of cortical bone, the outer layer of periosteum consists of dense irregular connective tissue, while the inner layer contains fibroblasts, osteoblasts, and osteoclasts 2-4. Periosteum is essential for bone growth, repair, and nutrition. It provides a surface for muscle and tendon attachment and contains nerves and blood vessels that supply oxygen and nutrients to the bone 5, 6. The periosteum's unique capacity to support bone regeneration stems from its stem cell niche, which responds to injury signals by activating downstream osteoblast precursors 7. During the lateral growth, periosteal osteoblasts drive bone formation on outer surfaces of cortical bone while osteoclasts resorb the endocortical surface, maintaining cortical thickness 8. In repair scenarios, periosteal stem cells are mobilized to differentiate into osteoblasts, contributing to callus formation following the initial resorptive phase by osteoclasts 9. However, the precise cross-talk between periosteal stem cells and osteoclasts during these processes remains an area of active investigation, particularly regarding the role of coupling factors within the periosteal microenvironment.

Osteoclasts are multinucleated cells that are derived from the monocyte/macrophage lineage and are found on the surface of bone tissue responsible for bone resorption, exhibit distinct distribution patterns and functional roles across different physiological contexts 10. During skeletal growth, osteoclasts are predominantly localized in the primary spongiosa of growth plates, where they resorb calcified cartilage to facilitate endochondral bone formation 11. This process is essential for longitudinal bone growth and proper skeletal morphogenesis. In contrast, under steady-state conditions, osteoclasts are sparsely distributed on endosteal and periosteal surfaces of cortical bone, with preferential localization in regions experiencing mechanical stress. Their activity is tightly coupled to osteoblast function through a complex network of coupling factors, including RANKL/RANK/OPG and ephrinB2-EphB4 signaling, which ensure balanced bone turnover 12. Emerging evidence suggests that disruptions in periosteal osteoclast contribute to skeletal pathologies 13. For instance, impaired osteoclast activity can lead to abnormal bone remodeling, while excessive resorption is associated with osteoporosis and fracture non-union 14-16. Understanding the nuanced differences in osteoclast behavior across growth, homeostasis, and repair contexts is therefore crucial for developing targeted therapies. Osteoclasts in bone marrow are mainly located in Howship's lacunae on the surface of trabecular bone 17. Osteoclasts in cortical bone are mainly located in the periosteum, and this group of periosteum-derived osteoclasts is closely involved in the growth, development and aging of bones 18. Unlike osteoclasts on the surface of trabecular bone in the bone marrow cavity, which exist in a multinucleated state, periosteum-derived osteoclasts in the cortical bone are predominantly in a mononuclear state 19. This disparity is likely resulting in distinct metabolic profiles of cortical bone and trabecular bone; however, the underlying mechanism of why periosteal osteoclasts remain mononuclear is still unknown.

Sialic acids (SAs) are a family of acidic sugars found abundantly in the human body, typically located on the outer surface of cell membranes and at the ends of glycans, which are sugar chains attached to proteins or lipids. SAs can be attached to galactose (Gal) or N-acetylgalactosamine (GalNAc) units via α2,3- or α2,6-linkages, on both N- and O-linked glycans 20. Our previous research demonstrated that α2,3-linked SA is crucial for osteoclast formation by mediating pre-osteoclast cell recognition 21. Mammalian sialidases, also known as neuraminidases (NEUs), are enzymes that remove sialic acid residues from glycan chains 22. There are four known types of sialidases in mammals, classified based on their localization and substrate specificity: NEU1, NEU2, NEU3, and NEU4, which exhibit distinct expression patterns in various tissues and subcellular distributions. NEU1 is the predominant isoform in extracellular, followed by NEU3, while NEU2 and NEU4 expressed minimally in intracellular 23. Dysregulation of sialidases has been implicated in various human diseases, including lysosomal storage disorders, cancer, neurodegenerative disorders, and viral infections 24-27.

Our study investigates the molecular mechanisms by which periosteal fibroblast-derived NEU1 modulates osteoclast activity, maintaining cortical bone integrity and promoting fracture healing. We hypothesize that targeted modulation of NEU1 activity can enhance bone regeneration and improve outcomes in conditions characterized by impaired bone healing. By exploring NEU1 as a potential therapeutic target, we aim to provide a foundation for novel strategies to accelerate fracture repair and improve bone health in clinical settings.

## Results

### Spatial differences in TRAP^+^ cell morphology and number in cortical and trabecular bone

**Figure 1:**
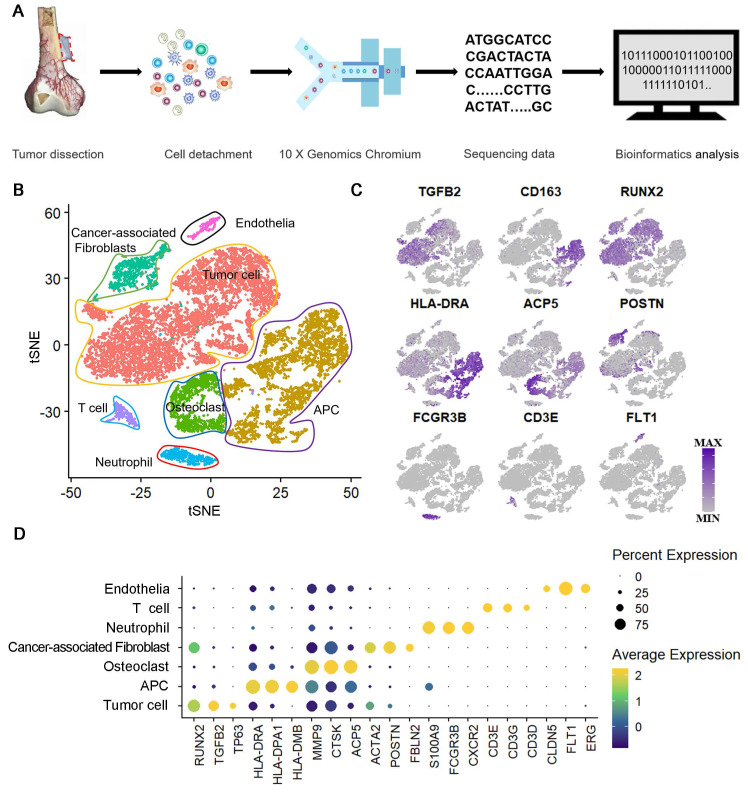
** Spatial differences in TRAP^+^ cell morphology and number in cortical and trabecular bone.** (A) Experimental design of bone tissue section observation; (B) Representative image of morphological differences between precursor osteoclasts (pOCs, red arrows) and mature osteoclasts (mOCs, blue arrows), scale bar = 100 μm; (C) Differential gene expression between pOCs and mOCs, n = 3; (D) Representative images of bone resorption pits from pOCs and mOCs groups, scale bar = 1 mm; (E) Quantification of bone resorption pits from pOCs and mOCs. (F-G) Representative TRAP staining of mouse distal femur, the area of interest shows the TRAP^+^ osteoclasts (red arrows) on cortical bone and trabecular bone, scale bar = 700 μm; (H) Quantification of TRAP^+^ osteoclasts on bone surface (BS), n = 5; (I) Quantification of TRAP^+^ activity per osteoclast, n = 5; (J) Quantification of TRAP^+^ mature osteoclasts on bone surface, n = 5; (K) Quantification of TRAP^+^ preosteoclasts on bone surface, n = 5. Data are presented as means ± SD. Statistical significance was performed through the utilization of an unpaired t-test **C, E, H-K**). *p < 0.05, **p < 0.01, N.S. = not significant.

To investigate the distinct roles of osteoclasts in cortical and trabecular bone, we performed tartrate resistant acid phosphatase (TRAP) staining on femurs and tibias from adult mice. We quantified the number and observed the morphology of osteoclasts in the periosteal (cortical bone) and trabecular (trabecular bone) regions (Figure 1A). Osteoclasts, derived from macrophages, can be classified as precursor osteoclasts (pOCs) or mature osteoclasts (mOCs) based on their differentiation status and the number of nuclei (Figure 1B). Previous studies indicate differences in gene expression and function between pOCs and mOCs 28, with pOCs exhibiting weak bone resorption and promoting osteogenesis via platelet-derived growth factor BB (PDGF-BB) secretion (Figure 1C-E). Our microscopic analysis revealed that periosteal osteoclasts in cortical bone are rare and predominantly mononuclear (Figure 1F), whereas trabecular osteoclasts are more abundant, multinucleated, and show higher TRAP activity (Figure 1G). We observed that multinucleated osteoclasts responsible for active bone resorption are primarily located in the trabecular region, while periosteal osteoclasts in cortical bone mainly remain in a mononuclear precursor state (Figure 1H-K). These findings align with previous studies 19, 28, underscoring the spatial differences in TRAP+ cell morphology and number, which play a crucial role in maintaining bone remodeling homeostasis in cortical and trabecular bone.

### NEU1 inhibits osteoclast differentiation, fusion and its histological distribution

**Figure 2:**
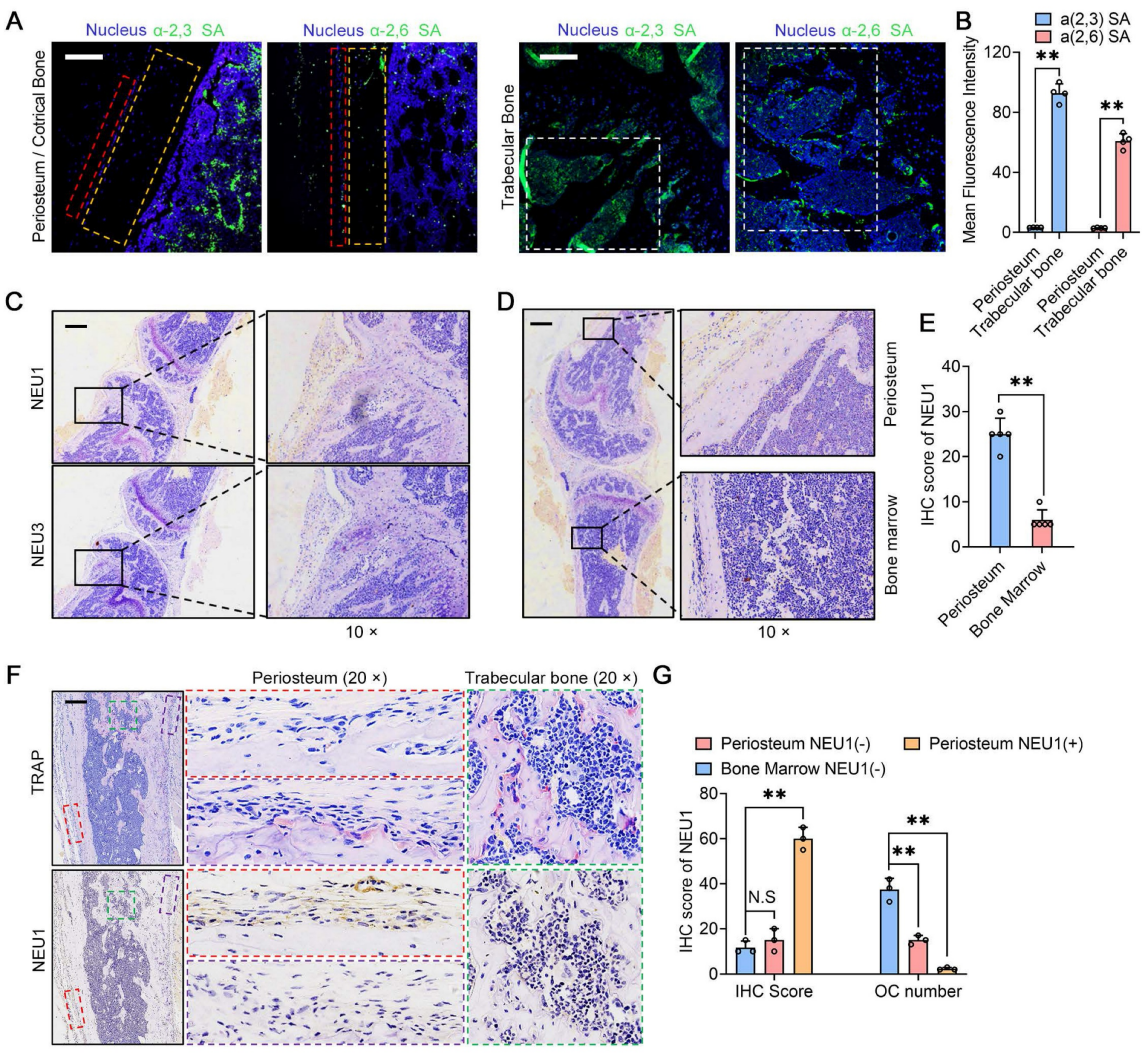
** NEU1 inhibits osteoclast differentiation, fusion and its histological distribution.** Representative α-2,3 SA and α-2,6 SA fluorescence staining in the periosteum and trabecular bone areas; scale bar = 150 μm in periosteum area; scale bar = 300 μm in trabecular area. Red box depicts periosteum area. Orange box depicts cortical area. White box depicts trabecular bone area. (B) Quantification of mean fluorescence intensity for α-2,3 SA and α-2,6 SA in the periosteum and trabecular bone, n = 4; (C) Representative IHC stain of NEU1 and NEU3 in the bone tissue, scale bar = 1 mm; (D) Representative IHC stain of NEU1 in the periosteum and bone marrow, scale bar = 1 mm; (E) Quantification of NEU1 IHC scores in periosteum and bone marrow, n = 5; (F) Representative IHC stain of TRAP and NEU1 in the periosteum and trabecular bone in continuous sections, scale bar = 1 mm. (G) Quantification of NEU1 IHC scores and osteoclast (OC) number in regions of interest. Data are presented as means ± SD. Statistical significance was performed through the utilization of an unpaired t-test (**B, E**), one-way ANOVA (**G**) followed by Student-Newman-Keuls post hoc tests. *p < 0.05, **p < 0.01, N.S. = not significant.

In our previous studies, we found that sialic acid is an important regulatory molecule for osteoclast recognition, fusion, and differentiation via mediating Siglec15-TLR2 binding on pre-osteoclasts 21. Masahiko Takahata *et al.* also highlighted the importance of local sialic acid levels in osteoclast differentiation 29. We performed immunofluorescence staining for α-2,3 SA and α-2,6 SA in cortical and trabecular bone regions. The results indicated that α-2,3 SA and α-2,6 SA expressions were minimal in the cortical periosteum but abundant in the bone marrow surrounding trabecular bone (Figure 2A-B). Considering that low sialic acid levels inhibit pre-osteoclast fusion and maturation, potentially regulated by NEU1 or NEU3, we examined the expression of these neuraminidases in the periosteal and trabecular regions. Immunohistochemistry (IHC) staining results showed that NEU1 was more highly expressed than NEU3 in bone tissue (Figure 2C). NEU1 was predominantly expressed in the periosteum but not in the bone marrow, highlighting its potential role in maintaining low sialic acid levels in cortical bone (Figure 2D-E, Figure S1). To confirm this, we performed IHC of TRAP and NEU1 in the same regions of cortical and trabecular bone to explore the correlation between osteoclast distribution and NEU1 expression. Interestingly, in cortical periosteum regions with high NEU1 expression, TRAP^+^ multinucleated osteoclasts were rarely observed, while TRAP+ mononuclear pre-osteoclasts were present in regions with lower NEU1 expression (Figure 2F). Conversely, the trabecular bone region, which had the lowest NEU1 expression, was enriched with TRAP^+^ multinucleated osteoclasts (Figure 2G). These findings indicate that the morphological and quantitative differences in osteoclasts between cortical and trabecular bone are potentially mediated by periosteal NEU1.

### Desialylation of cell surface glycoconjugates inhibits preosteoclast fusion recognition

**Figure 3:**
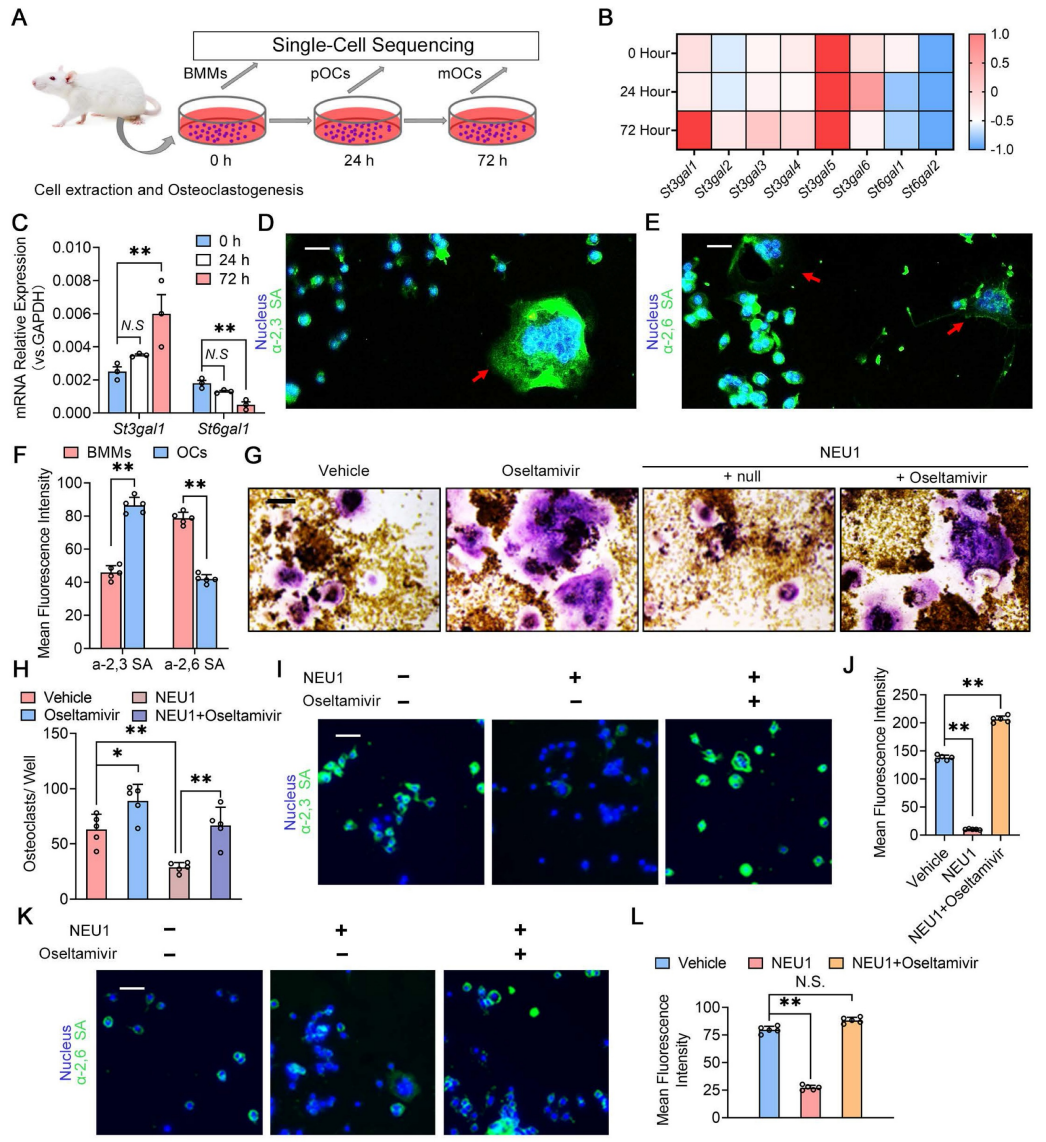
** Desialylation of cell surface glycoconjugates inhibit preosteoclast fusion recognition.** (A) Schematic representation of cell extraction and osteoclastogenesis from bone marrow monocytes (BMMs) to pOCs and to mOCs; (B) Heatmap showing the expression levels of various *St3gal* and *St6gal* genes during osteoclastogenesis; (C) Relative mRNA expression levels of *St3gal1* and *St6gal1* in osteoclastogenesis, n = 3; (D) Representative α-2,3 SA staining image of the BMMs and OCs, red arrows indicate OCs, scale bar = 50 um; (E) Representative α-2,6 SA staining image of the BMMs and OCs, red arrows indicate OCs, scale bar = 50 um; (F) Quantification of mean fluorescence intensity for α-2,3 SA and α-2,6 SA in BMMs and OCs; (G) Representative TRAP staining image of OCs treated with vehicle, oseltamivir, NEU1, or NEU1 + oseltamivir, scale bar = 100 um; (H) Quantification of osteoclasts per well in different treatment group, n = 5; (I) Representative fluorescence image showing α-2,3 SA in OCs treated with vehicle, oseltamivir, NEU1, or NEU1 + oseltamivir, scale bar = 50 um; (J) Quantification of mean fluorescence intensity for α-2,3 SA in OCs in different treatment group, n = 5; (K) Representative fluorescence image showing α-2,6 SA in OCs treated with vehicle, oseltamivir, NEU1, or NEU1 + oseltamivir, scale bar = 100 um; (L) Quantification of mean fluorescence intensity for α-2,6 SA in OCs in different treatment group, n = 5; Data are presented as means ± SD. Statistical significance was performed through the utilization of an unpaired t-test (**F, H**), one-way ANOVA (**C, J, L**) followed by Student-Newman-Keuls post hoc tests. *p < 0.05, **p < 0.01, N.S. = not significant.

To investigate the role of sialylation in osteoclast fusion and differentiation, we sequenced the transcriptome of osteoclasts at different stages and analyzed the expression of sialyltransferase genes during osteoclastogenesis (Figure 3A). The heatmap showed continuous upregulation of *St3gal1* and progressive reduction of *St6gal1* transcription levels during osteoclastogenesis (Figure 3B). Quantitative Real-time polymerase chain reaction (RT-qPCR) results were consistent with sequencing data, indicating the up-regulation of *St3gal1* and the down-regulation of *St6gal1* during osteoclastogenesis (Figure 3C). We then detected the expression of α-2,3 SA and α-2,6 SA on the osteoclastic membrane and found that α-2,3 SA and α-2,6 SA were all expressed during the differentiation of osteoclasts (Figure 3D-E). Notably, the expression level of α-2,3 SA increased with the multinucleation and maturation of osteoclasts, while α-2,6 SA expression decreased during the transition from mononuclear pre-osteoclasts to mature multinucleated osteoclasts (Figure 3F). To verify NEU1's ability to desialylate osteoclasts, we added recombinant NEU1 or oseltamivir to the osteoclastogenesis system and measured changes in α-2,3 SA and α-2,6 SA levels on osteoclasts. Oseltamivir, a specific neuraminidase inhibitor, effectively inhibits neuraminidase activity. We found that NEU1 significantly inhibited osteoclast multinucleation, while oseltamivir reversed this effect (Figure 3G-H). Immunofluorescence staining revealed that NEU1 effectively desialylated osteoclasts, significantly down-regulating α-2,3 SA and α-2,6 SA expression on the membrane surface (Figure 3I, K). Conversely, oseltamivir alleviated the desialylation effect of NEU1, restoring α-2,3 SA and α-2,6 SA expression (Figure 3J, L). Our previous study showed that sialylated toll-like receptor 2‌ (TLR2) binding to sialic acid-binding immunoglobulin-like lectin 15 (Siglec15) mediates cell recognition by cell fusion initiating osteoclast formation 21. The interaction of α-2,3 SA sialylated TLR2 with Siglec15 activates downstream signaling, promoting mononuclear pre-osteoclast fusion (Figure S2). Additionally, NEU1 hydrolyzes α-2,3 SA on osteoclasts, hindering TLR2 binding to Siglec15 and inhibiting osteoclast precursor fusion (Figure S3). These findings strongly suggest that sialylation is necessary for the recognition and fusion of osteoclast precursors, and NEU1 effectively desialylates the osteoclast surface, further inhibiting pre-osteoclast fusion.

### Single-cell RNA-seq identifies NEU1 enrichment in a unique fibroblast subpopulation within human periosteum

**Figure 4:**
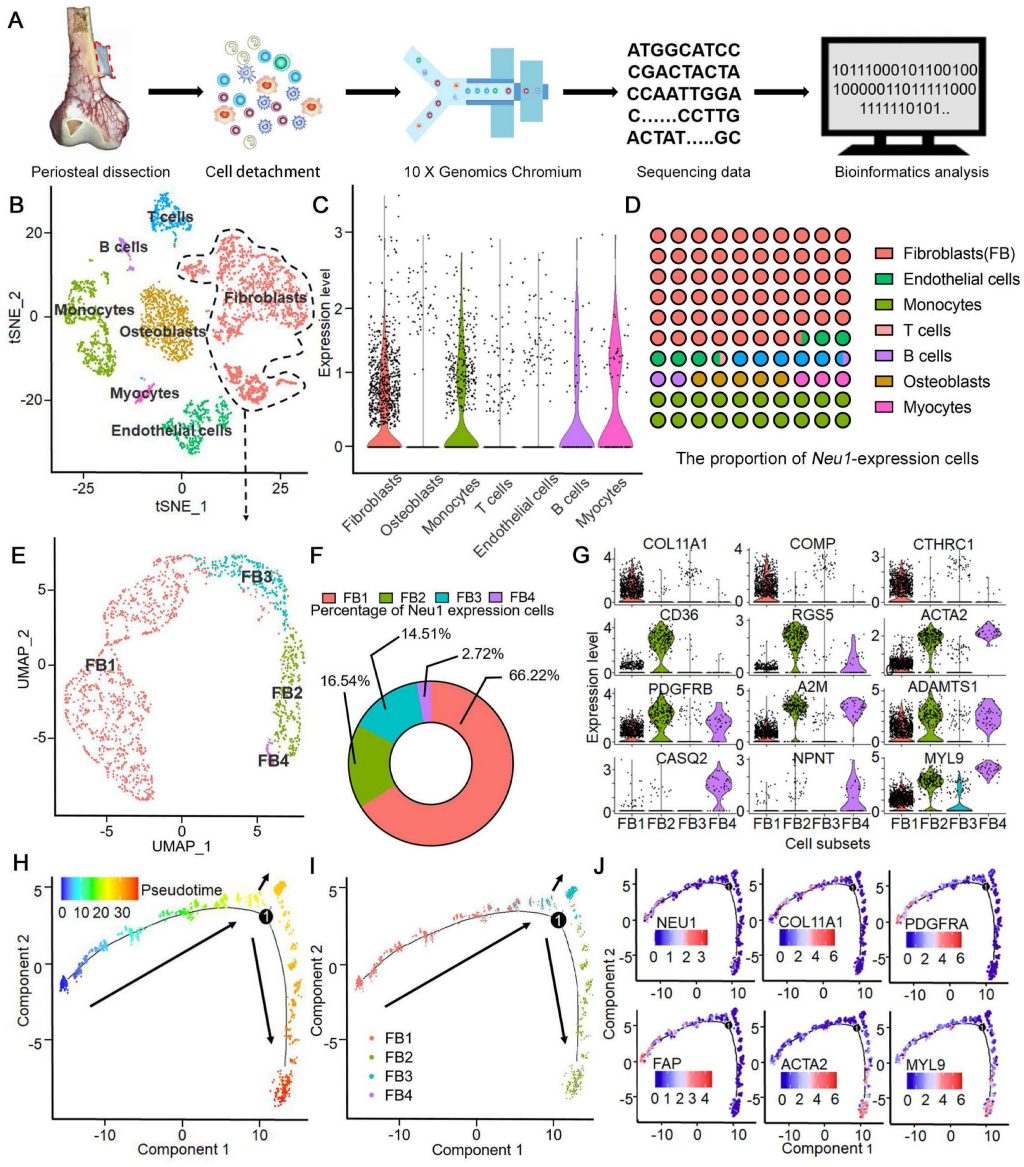
** Single-Cell RNA-Seq identifies Neu1 enrichment in a unique fibroblast subpopulation within human periosteum.** (A) Schematic diagram of periosteal cell extraction, single-cell RNA sequencing using 10X Genomics Chromium, and bioinformatics analysis; (B) tSNE plot showing distinct cell clusters from the periosteum, including fibroblasts, osteoblasts, myocytes, endothelial cells, monocytes, B cells, and T cells; (C) Violin plots showing *NEU1* expression levels across different cell types, with high enrichment in fibroblasts; (D) Proportion of *NEU1*-expressing cells within each cell type cluster; (E) UMAP plot identifying four subpopulations of fibroblasts (FB1, FB2, FB3, FB4); (F) Donut chart displaying the percentage of *NEU1*-expressing cells in each fibroblast subpopulation, with FB1 showing the highest expression; (G) Violin plots displaying marker gene expression for the four fibroblast subpopulations, including *COL11A1*, *COMP*, *CTHRC1*, *CD36, RGS5*, *ACTA2*, *PDGFRB*, *A2M*, *ADAMTS1*, *CASQ2*, *NPNT*, and *MYL9*; (H) Pseudotime analysis of periosteal fibroblasts indicating differentiation trajectories; (I) Pseudotime analysis of the four fibroblast subpopulations, showing their progression along the developmental trajectory; (J) Gene expression dynamics of key marker genes (*NEU1*, *COL11A1*, *PDGFRA*, *FAP*, *ACTA2*, and *MYL9*) along the developmental pseudotime.

To investigate the source of enriched NEU1 in the cortical periosteal region, we performed single-cell RNA sequencing on freshly isolated periosteal cells from a male subject. Cell mRNA libraries were prepared, sequenced using a 10x Genomics Chromium system, and subjected to quality filtering. This process yielded expression matrices for 4161 cells (Figure 4A). Dimensional clustering based on principal component analysis (PCA) identified seven distinct cell clusters within the periosteal cells (Figure S4).

Differentially expressed genes (DEGs) for each cluster were identified using the Wilcoxon rank-sum test (Figure S5). The cell types in these clusters were determined based on identified markers: 1) cluster 1 is *S100A4*^hi^/*FAP*^hi^ fibroblasts; 2) cluster 2 is *PECAM1*^hi^/*EMCN*^hi^ endothelial cells; 3) cluster 3 is *CD3E*^hi^ T cells; 4) cluster 4 is *CD22*^hi^ B cells; 5) cluster 5 is *PAX7*^hi^/*DEX*^hi^ myocytes; 6) cluster 6 is *CD14*^hi^/*CD68*^hi^ monocytes and 7) cluster 0 is *ALP*^hi^/*SP7*^hi^ osteoblasts (Figure 4B). Analysis of *NEU1* expression across the total cell population revealed that *NEU1* is predominantly enriched in cluster 1 cells, identified as fibroblasts (Figure 4C). These findings suggest that *NEU1* enrichment in cortical bone regions likely originates from fibroblasts residing in the periosteum (Figure 4D). Given the heterogeneity within fibroblasts, we further analyzed to identify a subpopulation of *S100A4*^hi^/*FAP*^hi^ fibroblasts with high *NEU1* expression (Figure 4E). Based on known cell markers and functional genes, fibroblast subtypes were annotated as follows: 1) *COL11A1*^hi^ fibroblasts (cluster FB1), expressing *COL11A1/COMP/CTHRC1*^hi^; 2) *CD36*^hi^ fibroblasts (cluster FB2), expressing *CD36/RGS5/ACTA2*^hi^; 3) *PDGFRβ*^low^ fibroblasts (cluster FB3), expressing *PDGFRβ/A2M/ADAMTS1*^low^; and 4) *MYL9*^hi^ fibroblasts (cluster FB4), expressing CASQ2/NPNT/MYL9^hi^ (Figure 4F-G). To understand the shared and distinct biological processes between different fibroblast subtypes, we performed pseudotime analysis of fibroblast subsets (Figure 4H). Cell trajectory analysis showed the differentiation trends among various subsets, with stem-cell-specific FB1 differentiating into FB2, FB3, and FB4 (Figure 4I). Based on the signature markers of each subset and the expression trajectories of the *NEU1* gene, we found that as *FAP*^hi^ fibroblasts (FB1) differentiate into *FAP*^low^ fibroblasts (FB2) or myofibroblasts (FB3 and FB4), *NEU1* expression is gradually downregulated (Figure 4J). This indicates that *FAP*^hi^ fibroblasts (FB1) would play a major role in regulating *NEU1* concentrations in the periosteal microenvironment. To emphasize and corroborate that NEU1 is mainly expressed in periosteal fibroblasts, we analyzed the published periosteal single-cell data (GSE249528) and the results showed that in this single-cell sample, *Neu1* was expressed in both *ZsGreen^hi^/tdTomato^hi^* cell subsets, which the authors identified as cambium-layer periosteal cells (CL-PCs) and fibrous-layer periosteal cells (FL-PCs) (Figure S9A). Although it was shown that *Neu1* was not restricted to the fibrous layer and cambium, the results still supported that *Neu1* was enriched in the periosteum, which was consistent with our conclusions (Figure S9B). In addition, we found the authors claim that a subset of cells marked as *Pi16*, a fibroblast marker, also correlates with *Neu1* expression. Our claimed marker genes (*Comp, Cthrc1 and Col11a1*) also showed a high correlation with the expression of *Neu1* in this dataset (Figure S9A). Taken together, the results from published single-cell data confirm our conclusion that *NEU1* in the periosteal region is mainly derived from a fibroblast subpopulation.

### Fibroblast-derived NEU1 regulates osteoclast fusion and activity

**Figure 5:**
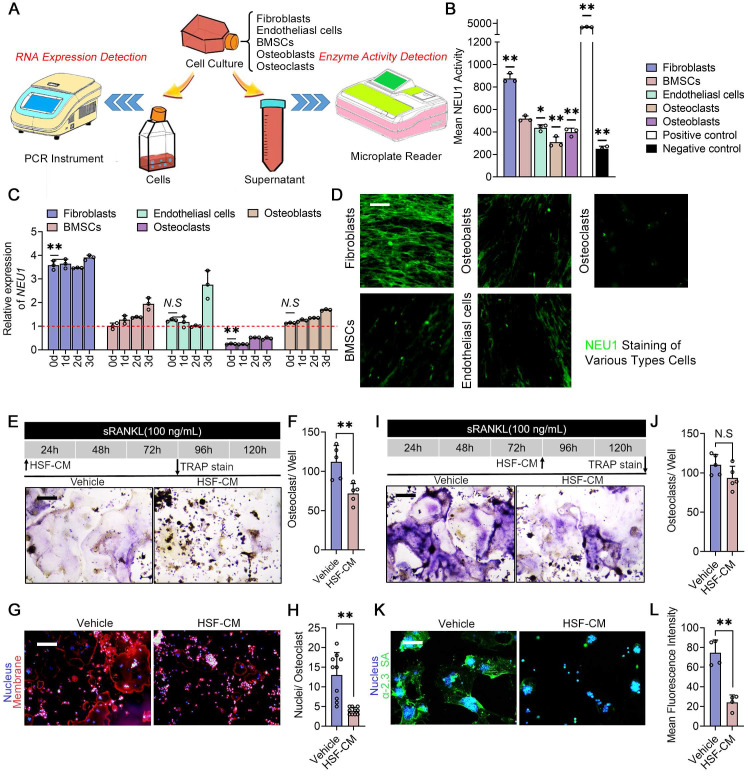
** Fibroblast-derived NEU1 regulates osteoclast fusion and activity.** Schematic diagram of the experimental design for *NEU1* RNA expression detection and NEU1 enzyme activity detection; (B) Mean NEU1 activity in fibroblasts, BMSCs, endothelial cells, osteoblasts, and osteoclasts, with positive and negative controls; (C) Relative expression of *NEU1* mRNA in fibroblasts, BMSCs, endothelial cells, osteoblasts, and osteoclasts over different time points, normalized to *GAPDH*, n = 3; (D) NEU1 staining in various cell types: fibroblasts, BMSCs, endothelial cells, osteoblasts, and osteoclasts, scare bar = 50 μm; (E) Representative TRAP staining of the OCs cultured with fibroblast conditioned medium (HSF-CM), scale bar = 100 μm. (F) Quantification of OCs per well, n = 5; (G) Representative fluorescent staining image of OCs cultured with HSF-CM, scale bar = 100 μm. (H) Quantification of mean nuclei number in OCs, n = 10; (I) Representative TRAP staining of the OCs which cultured with HSF-CM after maturation, scale bar = 100 μm. (J) Quantification of OCs per well, n = 5; (K) Representative sialic acid staining of OCs cultured with HSF-CM, scale bar = 100 μm. (L) Quantification of mean fluorescence intensity for α-2,3 SA in osteoclasts treated with vehicle or HSF-CM. Data are presented as means ± SD. Statistical significance was performed through the utilization of an unpaired t-test (**F, H, J, L**), one-way ANOVA (**B, C**) followed by Student-Newman-Keuls post hoc tests. *p < 0.05, **p < 0.01, N.S. = not significant.

To further support our notion that periosteal fibroblasts are the major source of localized high levels of NEU1 in the cortical bone region, we compared *NEU1* transcription and quantified NEU1 activity among periosteal resident cell populations, including fibroblasts, bone marrow mesenchymal stem cells (BMSCs), osteoblasts, osteoclasts, and endothelial cells (Figure 5A). Under the condition of total RNA consistency, RT-qPCR results indicated that fibroblasts exhibited the highest transcription levels of *NEU1* (Figure 5C). Moreover, NEU1 activity assays demonstrated that fibroblasts secreted the highest amounts of NEU1 into the medium compared to other cells, which was further confirmed by immunofluorescence staining (Figure 5B, D). To explore whether fibroblast-derived NEU1 could inhibit pre-osteoclast fusion, we added fibroblast-conditioned medium (HSF-CM) to the osteoclast induction system at different stages. TRAP staining revealed that the fibroblast-conditioned medium partially inhibited the formation of multinucleated osteoclasts but did not affect differentiation progression (Figure 5E-F, I-J). Average nuclear number statistics also illustrated that NEU1 from fibroblasts reduced the fusion capacity of osteoclast precursors, as indicated by fewer nuclei in mature osteoclasts from the HSF-CM group (Figure 5G-H). Meanwhile, we silenced the *Neu1* gene in fibroblasts for collecting conditioned medium and used it to culture pre-osteoclast. The results obtained were consistent with those of the oseltamivir group (Figure S10). Immunofluorescence staining further demonstrated the inhibitory effect of fibroblast-conditioned media on osteoclast sialylation, characterized by low levels of α-2,3 SA and α-2,6 SA detected on the cell membrane (Figure 5K-L, Figure S6). In summary, fibroblasts produce large amounts of NEU1 and interfere with osteoclast precursor fusion, playing a major role in regulating local sialic acid levels around periosteal and cortical bone regions.

### Sialidase inhibitors enhance periosteal osteoclast activity and reduce cortical bone thickness by increasing local sialic acid levels

**Figure 6:**
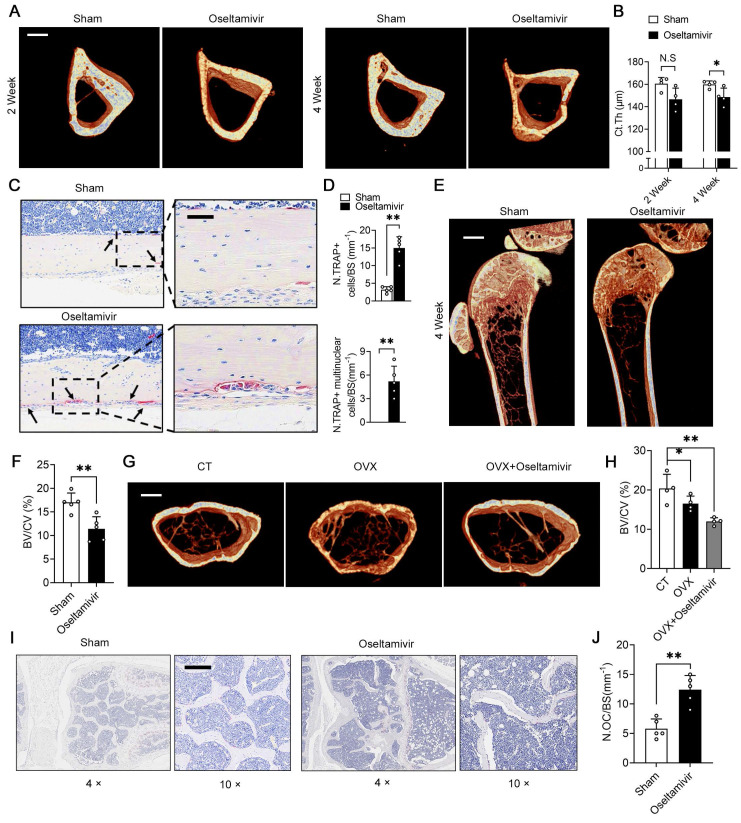
** Sialidase inhibitors enhance periosteal osteoclast activity and reduce cortical bone thickness by increasing local sialic acid levels.** (A) Representative micro-CT images of tibiae from sham and oseltamivir-treated mice at 2 and 4 weeks, scale bar = 500 μm; (B) Quantification of thickness of cortical bone of tibias (Ct.Th) from each group, n = 4; (C) Representative TRAP staining of cortical bone area, the area of interest is the periosteum, scale bar = 50 μm; (D) Quantification of TRAP^+^ osteoclasts and mature multinucleated osteoclasts, n = 5; (E) Representative micro-CT images showing trabeculae bone, scale bar = 1 mm; (F) Bone volume fraction (Bone volume/Total volume, BV/TV) analysis of trabeculae bone in control (CT) and ovariectomized (OVX) groups, n = 5; (G) Representative micro-CT images showing cortical and trabecular bone from three group mice; (H) BV/TV analysis of trabeculae bone in three groups, n = 4; (I-J) Representative TRAP staining of trabeculae bone area and quantification of the number of osteoclasts on bone surface (N.OC/BS), n = 5, scale bar = 200 μm; The data are shown as the means ± SD. Statistical significance was performed through the utilization of an unpaired t-test (**B, D, F, J**), one-way ANOVA (**H**) followed by Student-Newman-Keuls post hoc tests. *p < 0.05, **p < 0.01, N.S. = not significant.

To demonstrate that periosteal NEU1 is a key molecule in regulating pre-osteoclast differentiation and mediating cortical bone homeostasis, we performed NEU1 inhibition on young mice with active bone remodeling. We continuously fed 6-week-old mice with oseltamivir via gavage and collected tibias for micro-CT scans at Weeks 2 and 4. Micro-CT analysis revealed a decrease in cortical thickness of the tibia in oseltamivir-treated mice compared to the sham group, with a statistically significant difference observed at the fourth week of continuous oseltamivir feeding (Figure 6A-B). Furthermore, we observed the number and morphology of osteoclasts in the cortical bone regions and found a substantial increase in mononuclear and multinucleated TRAP+ osteoclasts in the cortical periosteum of oseltamivir-treated mice, a phenomenon rarely seen in wild-type adult mice (Figure 6C-D). Oseltamivir increases sialic acid levels in bone tissue by inhibiting NEU1 activity rather than its expression.

To further clarify NEU1's role in regulating cortical bone homeostasis, we assessed α-2,3 SA expression in bone tissue from the two groups. Immunofluorescence staining revealed that oseltamivir elevated sialic acid levels throughout the bone tissue, particularly reducing α-2,3 SA hydrolysis by periosteal NEU1 in cortical bone regions. This, in turn, promoted pre-osteoclast fusion and differentiation (Figure S7). We also evaluated the effect of sialic acid levels on cancellous bone mass. Micro-CT and statistical analyses indicated that the cancellous bone mass in sialidase inhibitor-fed young mice decreased by approximately 30% compared to control mice, suggesting that high sialic acid levels impair bone mass accumulation during bone remodeling (Figure 6E-F). To explore the effect of sialic acid levels on bone quality in osteoporotic mice, we performed ovariectomy on 12-week-old female mice and administered oseltamivir postoperatively. The rate of cancellous bone loss in oseltamivir-treated mice more than doubled compared to control mice, as evidenced by the assessment of cancellous bone mass (Figure 6G-H). This increased bone loss was associated with a higher number of active osteoclasts in bone tissue under high sialic acid conditions (Figure 6I-J). These results reveal that elevated sialic acid levels are detrimental to bone mass accumulation and cortical bone mechanical strength. In parallel, we also designed gain-of-function experiments to further validate and support the therapeutic value of NEU1 targeting. We established the OVX model to assess the effect of systemic NEU1 injection on bone loss rate, and administer NEU1 to fracture mice through localized injection for systematically evaluating indicator of fracture healing rate. After 4 weeks of the experiment, systemic administration of NEU1 partially slowed the rate of bone loss in OVX mice (Figure S11A). In mice with fractures, local NEU1 injection was detrimental to fracture healing, as evidenced by slow new bone formation and delayed medullary recanalization (Figure S11B). These *in vivo* experimental results provide direct evidence for the regulatory role of NEU1 in bone metabolism and fracture repair, thus further elucidating its therapeutic potential.

### Sialidase inhibitors enhance osteoclast activity and accelerate post-fracture callus remodeling

**Figure 7:**
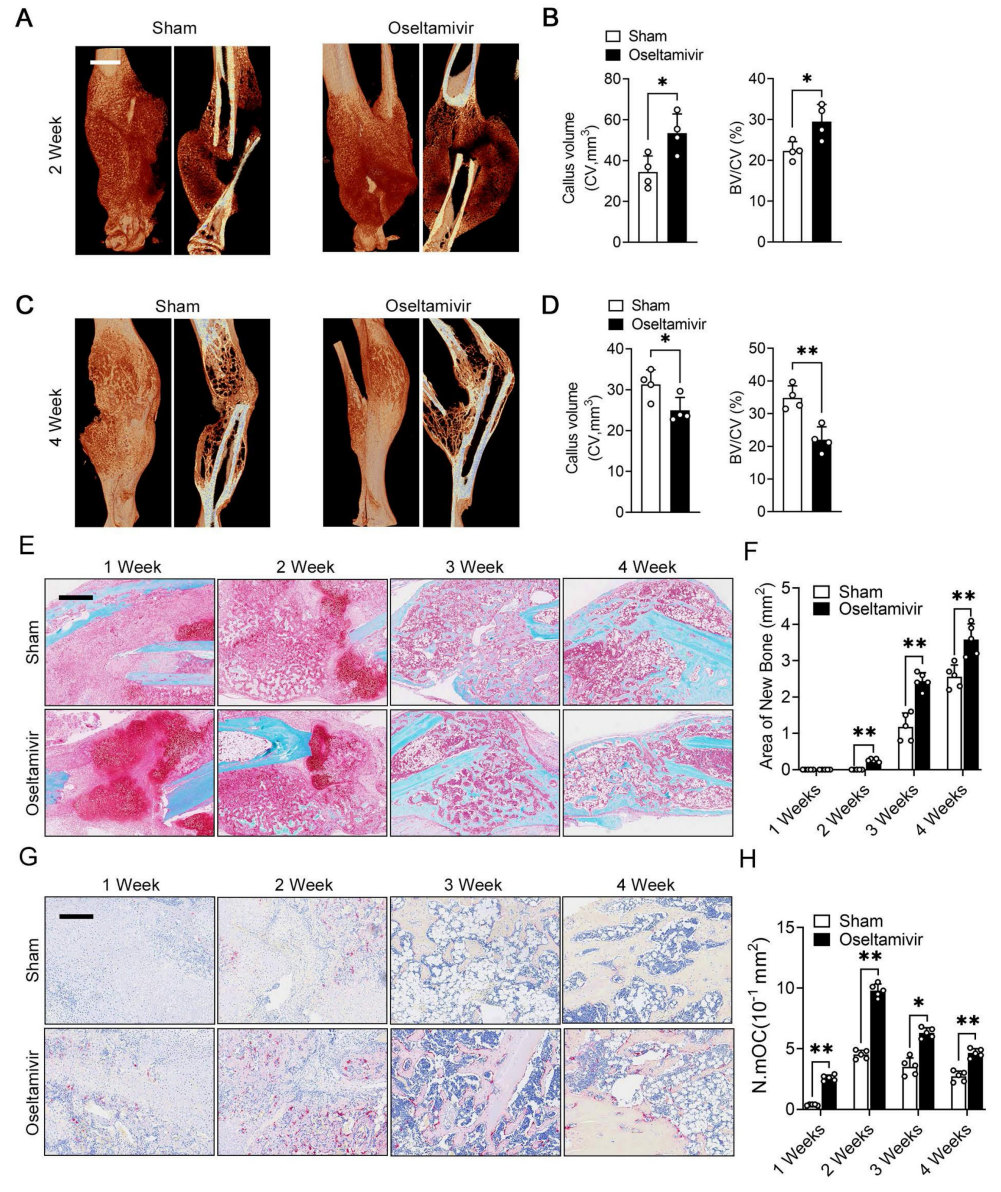
** Sialidase inhibitors enhance osteoclast activity and accelerate post-fracture callus remodeling.** (A-B) Representative micro-CT images showing callus located in the tibia at 2nd week, quantification of CV (callus volume) and BV/CV (Bone bone volume fraction) from each group, scale bar = 1.5 mm, n = 4; (C-D) Representative micro-CT images showing callus located in the tibia at 4th week, quantification of CV and BV/CV from each group, n = 4; (E-F) Representative Safranin O/Fast green staining images of fracture area, scale bar = 800 μm; Quantification of the area of reconstructed bone tissue from each group, n = 5. (G-H) Representative TRAP staining of fracture area, scale bar = 400 μm; Quantification of the number of mature osteoclasts (N.mOC) per unit area, n = 5. The data are shown as the means ± SD. Statistical significance was performed through the utilization of an unpaired t-test. *p < 0.05, **p < 0.01, N.S. = not significant.

The periosteum plays a crucial role in fracture healing, and our previous results indicated that periosteal fibroblast-derived NEU1 regulates cortical bone homeostasis. To explore the spatiotemporal function of fibroblast-derived NEU1 during fracture healing, we established a transverse fracture model in mice and interfered with NEU1 activity using oseltamivir. Micro-CT results showed that inhibiting NEU1 activity significantly altered the volume of early callus, characterized by a greater new bone tissue volume in the second week after fracture (Figure 7A-B). However, persistent inhibition of NEU1 activity accelerated the progression of bone remodeling in the late fracture repair stages, indicated by an earlier reopening of the medullary cavity and a smaller callus volume in the fourth week after fracture (Figure 7C-D). Safranin O/Fast green staining of the calli from both groups showed that oseltamivir intervention promoted the formation of calcified cartilage at the early stage (1 week) of fracture, with callus formation appearing in the second week, earlier than in the control group (Figure 7E). During callus formation and remodeling (3 and 4 weeks), the oseltamivir group exhibited better quality and quantity of new bone compared to the control group, achieving reopening of the medullary cavity earlier (Figure 7F). These observations suggest that inhibition of NEU1 activity may facilitate fracture repair and healing. To verify that the accelerated fracture healing due to inhibited NEU1 activity is associated with regulated osteoclast activity, we performed TRAP staining of calli at various stages. Results indicated that oseltamivir treatment activated osteoclasts at all stages of the callus, promoting osteoclast multinucleation and resorption, which contributed to early callus formation and late bone remodeling (Figure 7G-H). Additionally, staining the callus to assess NEU1 changes during fracture repair revealed that early in the fracture, a large number of fibroblasts enriched in NEU1 filled the break-end area, resulting in locally low levels of sialic acid (Figure S8). As fibroblasts underwent chondrogenic differentiation or regression, NEU1 levels in the callus decreased, and active multinucleated osteoclasts appeared. These findings demonstrate that fibroblast-derived NEU1 is temporally coupled with the transition from the fibrous junction to the callus stage during bone repair. Inhibiting NEU1 activity in the callus activates the physiological function of osteoclasts ahead of time, accelerating fracture repair.

## Discussion

Our study provides significant insights into the distinct metabolic profiles of cortical and trabecular bone by elucidating the role of periosteal NEU1 in regulating osteoclast activity and bone homeostasis. We demonstrated that NEU1, derived from periosteal fibroblasts, plays a critical role in preventing the fusion and multinucleation of osteoclast precursors in cortical bone. This action maintains osteoclasts in a mononuclear state, facilitating their anabolic functions and contributing to the low metabolic rate and unique structural characteristics of cortical bone compared to trabecular bone. Our findings highlight the importance of NEU1 in maintaining cortical bone integrity and offer new perspectives on bone metabolism and potential therapeutic approaches for bone-related diseases.

Traditionally, mechanical load was considered the primary factor regulating cortical bone homeostasis, with minimal involvement of osteoclasts. 30, 31. In the 1960s, E.A. Tonna first reported the presence of TRAP^+^ mononuclear osteoclasts on the surface of cortical bone critical for cortical bone growth and repair 32. Conflicting findings have since emerged regarding osteoclasts in different types of osteoporosis. In estrogen-deficient osteoporosis, the number of osteoclasts on trabecular bone surfaces increases 33, 34. Conversely, primary osteoporosis in the elderly shows a significant reduction in cortical bone osteoclasts, correlating with substantial cortical bone thickness loss 35, 36. These observations suggest that osteoclasts may have opposing roles in regulating cortical and trabecular bone homeostasis.

Studies have shown that periosteal osteoclasts outside of cortical bone exhibit a mononuclear characteristic 19, 28. Unlike bone marrow osteoclasts, which fuse to form multinucleated syncytia, periosteal osteoclasts rarely fuse and remain in a precursor state, aligning with the low turnover rate of cortical bone. Initially, the disparity in multinucleation was thought to be due to local RANKL/OPG levels 37, 38. However, our findings indicate varying sialylation levels, due to distinct NEU1 enrichment, as the key factor regulating the fusion and multinucleation of osteoclasts. Mononuclear TRAP^+^ osteoclasts rarely resorb bone but have pro-anabolic functions, releasing PDGF-BB to stimulate osteogenesis and angiogenesis through interactions with osteoblasts and *CD31*^high^*Emcn*^high^ endothelial cells 39-41. This anabolic effect helps maintain the low metabolic rate of cortical bone compared to trabecular bone, leading to differences in mechanical strength and structural characteristics to meet specific physiological needs. A recent study by Liu *et al.* 42, sheds light on the role of fibrous-layer periosteal cells (FL-PCs) in bone healing and remodeling. The study demonstrated that FL-PCs do not contribute to steady-state osteogenesis but form the primary structure of fibrocartilaginous callus during fracture healing. Post-fracture, FL-PCs invade the cambium-layer periosteum and bone marrow, forming neo-skeletal stem/progenitor cells (SSPCs) that maintain healed bones into adulthood.

Differences in the multinucleation and function of osteoclasts between cortical and trabecular bone can lead to opposite trends in their numbers during osteoporosis progression. Osteoporosis, characterized by progressive bone mass loss, shows varied patterns between cortical and trabecular bone. In estrogen-deficient osteoporosis, osteoclast numbers increase on trabecular bone surfaces due to more multinucleated osteoclasts and their heightened catabolic activity. Conversely, primary osteoporosis shows a reduction in periosteal osteoclasts on cortical bone surfaces, primarily due to fewer TRAP^+^ mononuclear osteoclasts and diminished anabolic effects 43. These differences underscore the need for tailored therapeutic approaches for osteoporosis, considering the specific osteoclast changes in cortical and trabecular bone. Congenital NEU1 deficiency results in sialidases, a severe lysosomal storage disorder with a broad spectrum of clinical manifestations, including skeletal deformities, muscle hypotonia, and weakness 44, 45. *Neu1-KO* zebrafish exhibit reduced body length and weight compared to wild-type fish, with adults showing vertebral curvature. These zebrafish also display decreased expression of genes involved in bone remodeling, mirroring the phenotypes seen in *Neu1-KO* mice and human sialidosis patients 46-48. This consistency across species highlights the critical role of NEU1 in skeletal health and bone remodeling.

The periosteum is vital for the growth, modeling, and healing of cortical bone. While trabecular bone remodels within the bone marrow microenvironment, the periosteum oversees cortical bone growth and modeling 6. In young bones, the periosteum is thick and vascularized, but it thins and loses vascularity with age 49, 50. Loss of the periosteum due to injury or disease often results in weakened or non-functional bones. Expanding cortical bone through periosteal activity can significantly enhance bone strength, regardless of bone mass density 7, 51, 52. Recent studies highlight the crucial role of periosteal fibroblasts in cortical bone homeostasis, though the specific molecular mechanisms are unclear 37, 53, 54. Our single-cell RNA sequencing of human periosteum identified a unique fibroblast subgroup with elevated NEU1 levels. This NEU1 activity removes SAs, preventing the fusion and maturation of osteoclast precursors in the periosteum. We demonstrated that periosteal fibroblasts maintain osteoclast precursors in a mononuclear state through NEU1 secretion, enabling their anabolic functions and regulating cortical bone homeostasis. Targeting NEU1 activity in the periosteum improved fracture healing in mouse models.

This study offers valuable insights into the mechanisms driving differences in cortical and trabecular bone homeostasis, providing a theoretical foundation for improving bone quality and reducing fracture risk in clinical practice. Our findings reveal that inhibiting NEU1 activity in the callus activates osteoclast functions, accelerating bone fracture repair. By advancing our understanding of the molecular mechanisms regulating bone metabolism, we can develop more effective treatments for osteoporosis and related fractures, ultimately enhancing patient outcomes in clinical practice.

## Materials and Methods

### Reagents

Mouse macrophage RAW264.7 cell line, HUVEC cell line, and HSF-1 cell line were obtained from the American Type Culture Collection (Rockville, MD, USA). Dulbeccos Modified Eagle Medium (DMEM, #PM150210, High D-glucose with 4500mg/L) and penicillin-streptomycin (#PB180120,100x) were purchased from Procell Life Science (Wuhan, China). Ftal bovine serum (FBS) was purchased from CELLCOOK (#CM1003, Guangzhou, China). Recombinant mouse RANKL were purchased from R&D Systems (#462-TEC-010/CF, Minneapolis, MN, USA). TRAP stain kit was obtained from Sigma Aldrich (#387-A, Shanghai, China). Rabbit anti-mouse NEU1 (#A6299, 1:100 dilution for IHC) and NEU3(#A13842, 1:50 dilution for IHC) were purchased from ABclonal (Wuhan, China). Oseltamivir was purchased from Roche (#H20140344, Shanghai, China). Sialidase also named neuraminidase was purchased from Merck (#N2876-25UN, Shanghai, China). NEU1 homologous protein was purchased from Merck (#11585886001, Roche, Switzerland). Biotinylated Maackia Amurensis Lectin II (MAL II, #B-1265-1) and Sambucus Nigra Lectin (SNA, #B-1305) were purchased from Vectorlabs (San Francisco, CA, USA).

### Histological TRAP and IHC evaluation

Bone tissue samples from mouse hind limbs were collected and fixed in 10% neutral-buffered formalin. After decalcified in 10% EDTA for 2 weeks, samples were embedded in paraffin and then sliced to 5 μm for subsequent TRAP and IHC staining. For histological TRAP stain, sections were incubated with TRAP stain solution (Sigma Aldrich, Shanghai, China) according to the manufacturers' instructions after gradient dewaxing. For IHC assessment, sections were first gradient dewaxed, washed, blocked with 3% H_2_O_2_ and immunostaining blocking fluid (#P0100A, Beyotime Biotechnology, Shanghai, China) for 30 min. Afterwards, diluted primary antibody was used to incubated sections at 4 °C for 12 h and then horseradish peroxide linked secondary antibody was used to incubated sections at 37 °C for 1 h. After incubation, the sections were washed and reacted with DAB Horseradish Peroxidase Color Development Kit (#P0203, Beyotime Biotechnology, Shanghai, China). Finally, nuclei were counterstained with hematoxylin (#C0105S, Beyotime Biotechnology, Shanghai, China), and the stained sections were mounted for subsequent observing and analyzing via German semi-quantitative scoring system. The scoring was conducted based on staining intensity and positive staining area. Both evaluations were performed under a light microscope. For each section, 5 non-overlapping representative fields of view were randomly selected, and the average value was calculated for the final computation. The semi-quantitative score of a specific field of view was obtained by multiplying the staining intensity score and positive staining area score of that same field. The scoring process was independently carried out by two experienced pathologists using the double-blind method.

### Immunofluorescence staining

For sialic acid staining, sections were subjected to gradient dewaxing, washing, and blocking, followed by incubation with biotinylated-MAL II (1:200 dilution) or biotinylated-SNA (1:100 dilution) overnight at 4 °C. After 3 times washed with PBS, sections were incubated with FITC-linked Streptavidin (#405202, Biolegend, California, USA) for 1 h at room temperature without light, after which nuclei were colored using DAPI (#C1005, Beyotime Biotechnology, Shanghai, China). Sections were observed and photographed by Leica immunofluorescence imaging system, and the fluorescence intensity was analyzed by image J v1.7. For sialic acid staining of osteoclasts, cells were fixed with 4% paraformaldehyde for 30 min and then washed and blocked with 5% BSA for 30 min. After blocking, cells were incubated with biotinylated-MAL II (1:200 dilution) or biotinylated-SNA (1:100 dilution) overnight at 4 °C and then incubated with FITC-linked Streptavidin for 1 h. Subsequently, cells were washed by PBS and counterstained with DAPI for 5 min. Images were obtained by Leica immunofluorescence imaging system.

### Osteoclast differentiation

RAW264.7 cells were cultured with osteoclastogenesis medium consisting of DMEM, 10% FBS, 1% penicillin-streptomycin, and 100 ng/mL RANKL to generate osteoclasts. Conditioned medium (CM) of fibroblasts was added into the induction medium (1:4) to culture osteoclasts. To inhibit NEU1 function in the CM of fibroblasts, we used the NEU1-targeted inhibitor oseltamivir in a concentration of 0.5 mM. The culture medium was replaced every 2 days and cells were incubated at 37 °C with 5% CO_2_. For TRAP staining, cells were fixed in 4% paraformaldehyde for 20 min and then stained with TRAP staining solution (#387-A, Sigma Aldrich, Shanghai, China) according to the manufacturer's instructions. For fluorescence staining, blocked osteoclasts were incubated with rabbit anti-mouse focal adhesion kinase antibody (#ab81298, Abcam, Hangzhou, China, 1:200 dilution) for 2 h at room temperature followed by 1 h with TRITC-linked secondary antibody (#ab6718, Abcam, Hangzhou, China, 1:1000 dilution). After DAPI staining of nuclei, images were obtained by Lycra microscopy.

### High-throughput sequencing analysis of periosteal tissue

Periosteal tissue was stored in tissue preservation solution (Miltenyi Biotec) until treatment. The sample was first washed with PBS and cut into small pieceson ice, and stirred for 50 min at 37 ° C with 150 U/mL Collagenase II (Worthington) and 2 mg/mL Collagenase IV (Worthington) and 1.2 U/mL Disase II (Worthington) and 50 U/mL DNaseI. After digestion, samples were screened through a 70 μm cell filter and centrifuged at 300 g for 5 min. After the supernatant was removed, the precipitated cells were suspended in red blood cell lysis buffer (Miltenyi Biotec) to lyse red blood cells. After washing with PBS containing 0.04% BSA, cell pellets were resuspended in PBS containing 0.04% BSA and re-filtered through a 35 μm cell filter. The scRNA-Seq libraries were generated using the 10X Genomics Chromium Controller Instrument and Chromium Single Cell 3' V3 Reagent Kits (10X Genomics, Pleasanton, CA). All libraries were sequenced by illumina sequencer (Illumina, San Diego, CA) on a 150 bp paired-end run. Fast filtering was applied to adapter sequences with default parameters and deletion of low-quality reads, and reads were aligned with the mouse genome with Cellranger v3.1.0 to obtain a characteristic barcode matrix. The samples were sorted according to the barcode reading of each sample, and the samples were analyzed down to get the aggregation matrix. We employed RStudio software for data analysis. For Pseudo-Time analysis, we preformed the Single-Cell Trajectories analysis utilizing Monocle3 package.

### siRNA transfection

Fibroblasts were cultured in complete DMEM (supplemented with 10% FBS and 1% penicillin-streptomycin) at 37 °C with 5% CO₂. For transfection, fibroblasts were seeded into 6-well plates at a density of 2×10⁵ cells/well and incubated for 24 h until reaching 70-80% confluence. According to the Lipofectamin2000 (#11668019, Thermo Fisher, USA) manufacturer's protocol, transfection complexes were prepared and transfection was performed. siRNA for mouse-*Neu1* were designed and purchased from MedChemExpress (#HY-RS18959, New Jersey, USA).

### qPCR analysis

Total RNAs from cells were extracted using rapid RNA extraction kit (#RN001, YISHAN Technologies, Shanghai, China) according to the manufacturer's instructions. Quantity and concentration of the RNA samples were measured by spectrometer (BioDrop µLite, Cambridge, England). For real-time PCR, the cDNA was synthesized using PrimeScript^TM^RT reagent kit (#DRR037A, Takara, Nojihigashi, Japan). SYBR Green Realtime PCR Master Mix (#QPK-201, TOYOBO, Japan) was used to perform real-time PCR. The light spectrum of SYBR was detected on a CFX96™ Real-Tim PCR System instrument (Bio-Rad). The primers used were listed in supplementary materials (Table S1).

### NEU1 activity assay

For the NEU1 activity assay, we used a neuraminidase assay kit (#P0306, Beyotime, Shanghai, China). Specifically, a 70 mL neuraminidase detection buffer was added to each well of the 96-well plate. In the control group, 0 or 10 mL of neuraminidase were added to each well respectively. Another 10 mL of neuraminidase solution were added to each well. Finally, add 0 or 10 mL Milli-Q water per well to give a total volume of 90 mL per well.

### Animal experiments

The fracture model was established in 8-week-old male mice. Mice were anesthetized with a mixture of oxygen and bromoethane, and then closed transverse fractures of the tibia were created using a three-point bending technique. Subsequently, fracture was confirmed by radiological examination. The tibias and femurs were dissected and extracted for subsequent experiments. To investigate the regulatory role of oseltamivir in cortical bone homeostasis, 4-week-old C57BL/6 mice were administered oseltamivir via intragastric gavage at a dose of 25 mg/kg body weight. The treatment was performed once every 3 days, with a continuous duration of 4 weeks. For the assessment of cortical bone quality, micro-computed tomography scans were conducted on the hindlimbs of the mice at the 2nd and 4th weeks of the experiment. For gain-of-function experiments, ovariectomized mice received systemic administration of NEU1 homologous protein via intraperitoneal injection at a dose of 0.2 U per mouse, once every 3 days. For fractured mice, 0.05 U of NEU1 homologous protein was injected into the fracture site once every 2 days. The control group was injected with an equal volume of PBS at the same site. All mice were fed in the animal facility of Third Military Medical University and the Institutional Animal Care and Use Committee of the Third Military Medical University reviewed and approved all experimental protocols.

### Micro-CT analysis

Tibia and femur specimens were fixed overnight in 4% paraformaldehyde. Specimens were scanned using computed tomography (skyscan 1272, Bruker microCT, Kontich, Belgium). The scanning accuracy was set to 8 μm and the data was reconstructed by NRecon v1.6. CTan v1.9 and CTvol v2.0 were used to analyze and visualize the reconstructed results.

### Safranin O-Fast green staining

The sections were dewaxed and washed 3 times with PBS, and then stained with fast green for 30 s. Washed 3 times with PBS, sections were differentiated with 1% alcohol hydrochloride for 10 s, and then stained with safranin O for 1 min.

### Statistical analysis

All data are presented as means ± SD. Comparisons between two groups were analyzed using independent unpaired two-tailed Student's t-tests, and one-way ANOVA followed by Student-Newman-Keuls post hoc tests are used for multiple comparisons. The *p* value < 0.05. was considered to be significant. * (*p* < 0.05) or ** (*p* < 0.01). was used to indicate the statistically significant differences between the treatment and control groups.

## Supplementary Material

Supplementary figures and table.

## Abbreviations

BMSCs: Bone marrow mesenchymal stem cells
DEGs: Differentially expressed genes
HSF-CM: Fibroblast-conditioned medium
IHC: Immunohistochemistry
mOCs: Mature osteoclasts
NEUs: Neuraminidases
NEU1: Neuraminidase
GalNAc: N-acetylgalactosamine
pOCs: Precursor osteoclasts
PCA: Principal component analysis
PDGF-BB: Platelet-derived growth factor BB
RT-qPCR: Quantitative Real-time polymerase chain reaction
Sas: Sialic acids
Siglec15: Sialic acid-binding immunoglobulin-like lectin 15
TLR2: Toll-like receptor 2‌
TRAP: Tartrate resistant acid phosphatase
