# Supplementary material for: Fibroblast-derived NEU1 as a therapeutic target for improving cortical bone integrity and fracture healing
Source: Theranostics. 2026 Jan 1;16(4):2101–17. doi: 10.7150/thno.119200 (PMC12680898; doi:10.7150/thno.119200)
Supplement: Supplementary file 1 — Supplementary figures and table. [file thnov16p2101s1.pdf]

# SUPPLEMENTARY MATERIALS

**Table S1** List of primers used for qRT-PCR

| **Gene names** | **Primer** | **Sequence** |
|---|---|---|
| Mouse *Neu1* | Forward | 5'-CGAATTGTCCTCCGCAGCTA-3' |
| Mouse *Neu1* | Reversed | 5'-TCCGCCATGAGGTACCATTG-3' |
| Mouse *Acp5* | Forward | 5'-AGACGAGGTTACGCTGTGC-3' |
| Mouse *Acp5* | Reversed | 5'-TCGGGGACAATTCGGTAAACT-3' |
| Mouse *Ctsk* | Forward | 5'- GCGGCATTACCAACAT -3' |
| Mouse *Ctsk* | Reversed | 5'- CTGGAAGCACCAACGA -3' |
| Mouse *Nfatc1* | Forward | 5'-GAGGAGTTGGCTCAGTG-3' |
| Mouse *Nfatc1* | Reversed | 5'-TAGCGTTCCGTTCGTT-3' |
| Mouse *OCstamp* | Forward | 5'- TTATGTGTTTCCACGAAGCCCTA -3' |
| Mouse *OCstamp* | Reversed | 5'- ACAGAAGAGAGCAGGGCAACG -3' |
| Mouse *Pdgfb* | Forward | 5'-CATCCGCTCCTTTGATGATCTT -3' |
| Mouse *Pdgfb* | Reversed | 5'-GTGCTCGGGTCATGTTCAAGT -3' |
| Mouse *St3gal1* | Forward | 5'- TCCAACACGGGAGTACCCA -3' |
| Mouse *St3gal1* | Reversed | 5'- GCTGGTCGAACCAATATGATACC -3' |
| Mouse *St6gal1* | Forward | 5'-CTCCTGTTTGCCATCATCTGC -3' |
| Mouse *St6gal1* | Reversed | 5'-GGGTCTTGTTTGCTGTTTGAGA -3' |
| Mouse *Gapdh* | Forward | 5'- AACGTCGGAACGATAGCA -3' |
| Mouse *Gapdh* | Reversed | 5'- CTTAAGGCTAGTTAGGCCC -3' |

**Figure S1**

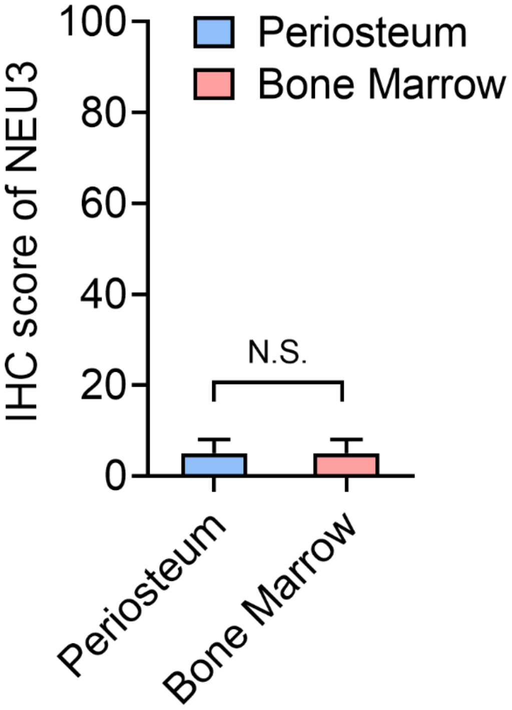


**Figure S1.** NEU3 IHC score of periosteum and bone marrow; n=3;

**Figure S2**

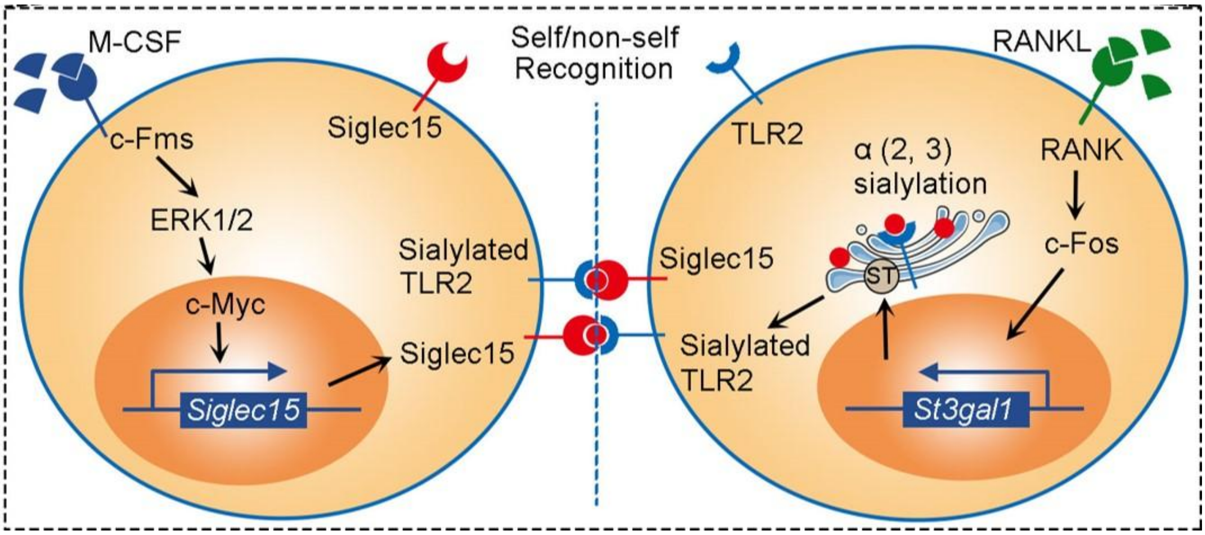


**Figure S2.** Schematic of Siglec15 mediating osteoclast recognition and fusion by binding to α-2,3 sialylated TLR2.

**Figure S3**

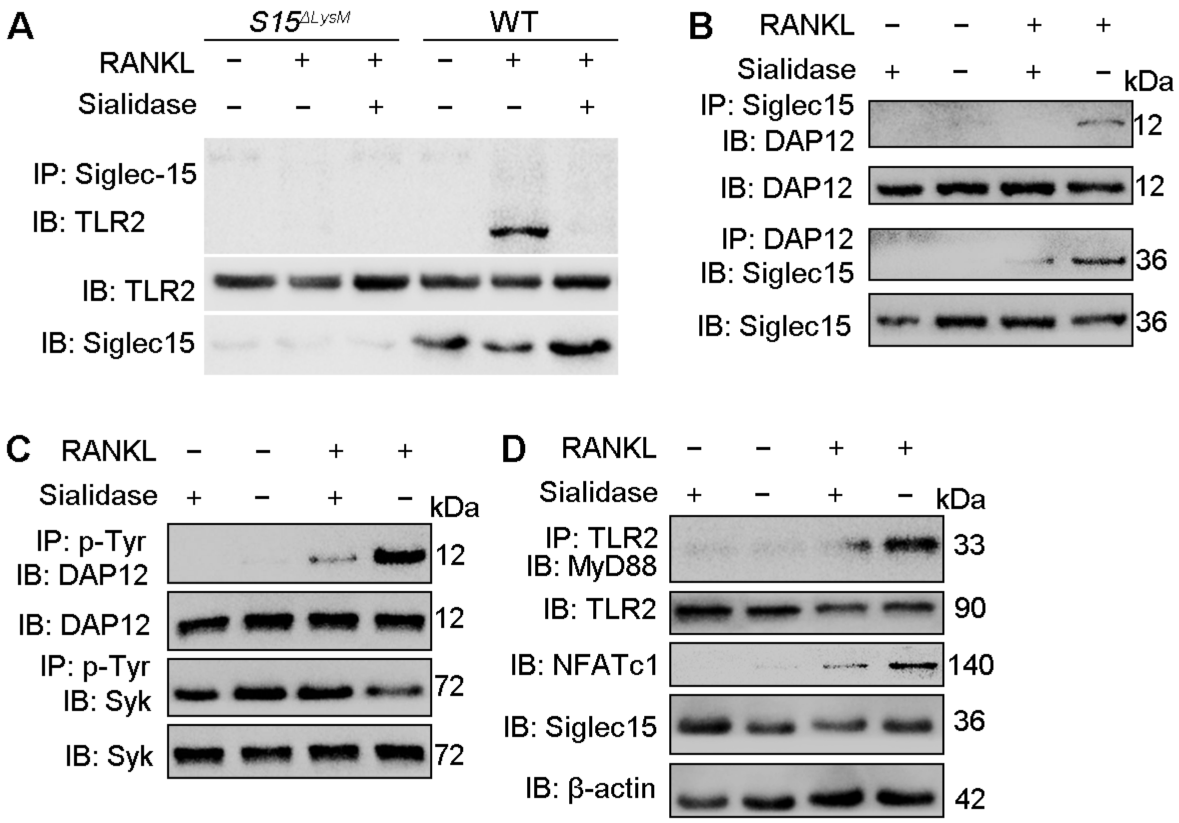


Image source : Ce Dou., et al., *Sialylation of TLR2 initiates osteoclast fusion. Bone Res*, 2022 Mar 2;10(1):24.

**Figure S3.** Sialic acid A-2,3 mediates the fusion of mononuclear osteoclasts.

(A) Immunoblotting (IB) for TLR2 in whole-cell lysates or protein complexes immunoprecipitated (IP) with Siglec15 in Siglec15ΔLysM or Siglec15fl/fl (WT) BMMs to test the effects of sialidase.

(B) IB for DAP12 in whole-cell lysates or protein complexes IP with Siglec15 and IB for Siglec15 in whole-cell lysates or protein complexes IP with DAP12 in the indicated groups.

(C) IB for DAP12 and Syk in whole-cell lysates or protein complexes IP with p-Tyr in the indicated groups.

(D) IB for MyD88 in protein complexes IP with TLR2 in the indicated groups. IB for TLR2, NFATc1, and Siglec15 in whole-cell lysates.

**Figure S4**

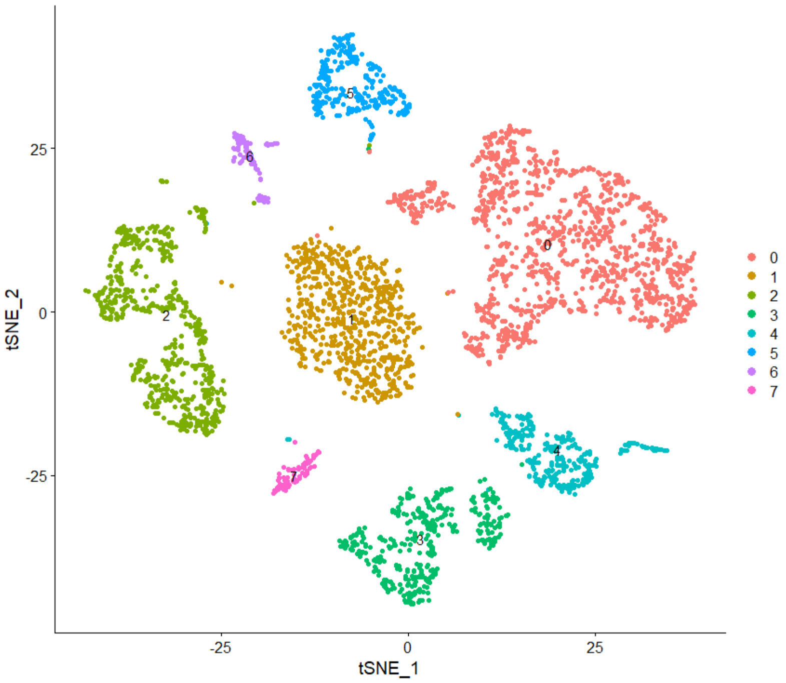


**Figure S4.** Clusters of cells that has passed through a reduced-dimension cluster

**Figure S5**

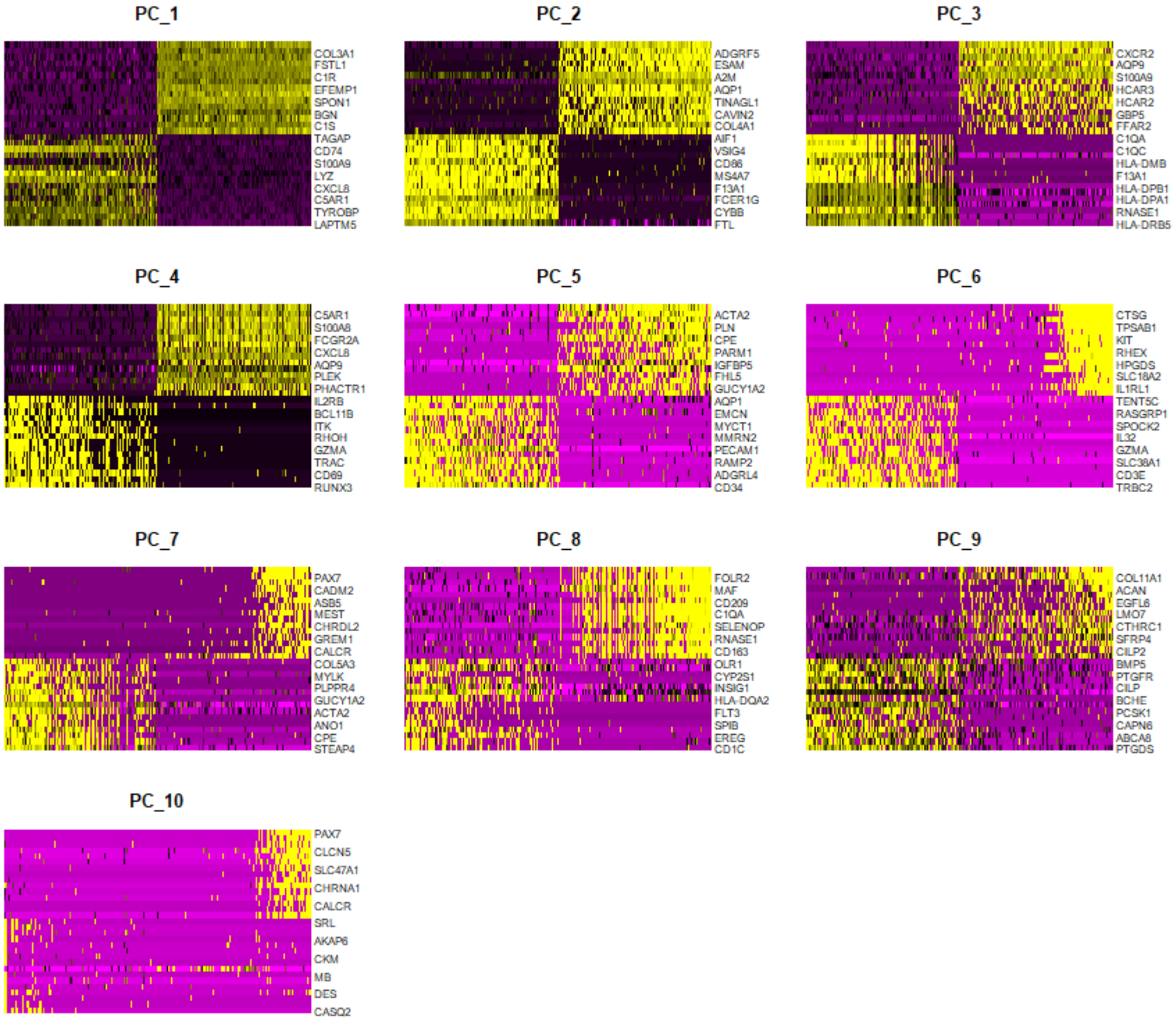


**Figure S5.** Differentially expressed genes (DEGs) of each cluster were identified with the Wilcoxon rank-sum test.

**Figure S6**

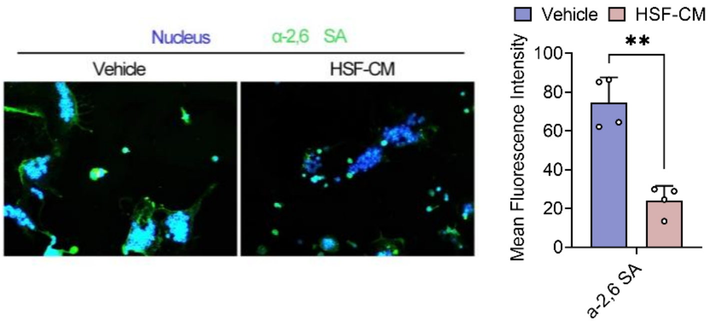


**Figure S6.** Immunofluorescence staining showed the inhibitory effect of fibroblast conditioned medium on the sialylation of osteoclasts (a-2,6 SA).

**Figure S7**

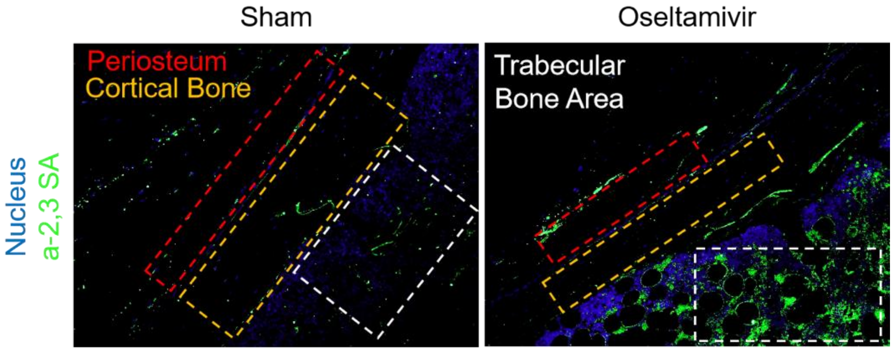


**Figure S7.** Oseltamivir effectively inhibits NEU1 activity and increases sialic acid levels in bone tissue

**Figure S8**

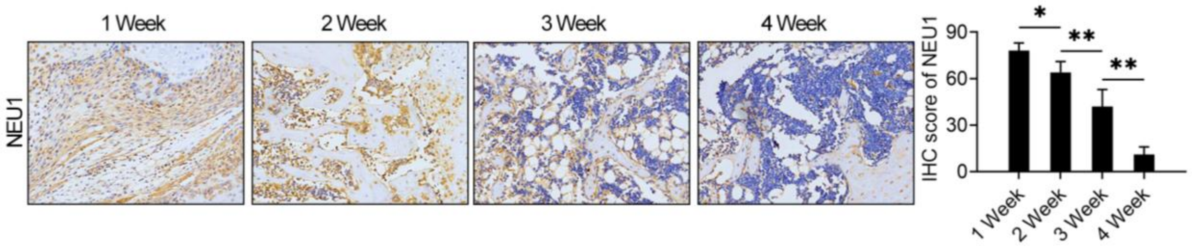


**Figure S8.** Expression level of NEU1 in callus at different stages of fracture healing.

**Figure S9**

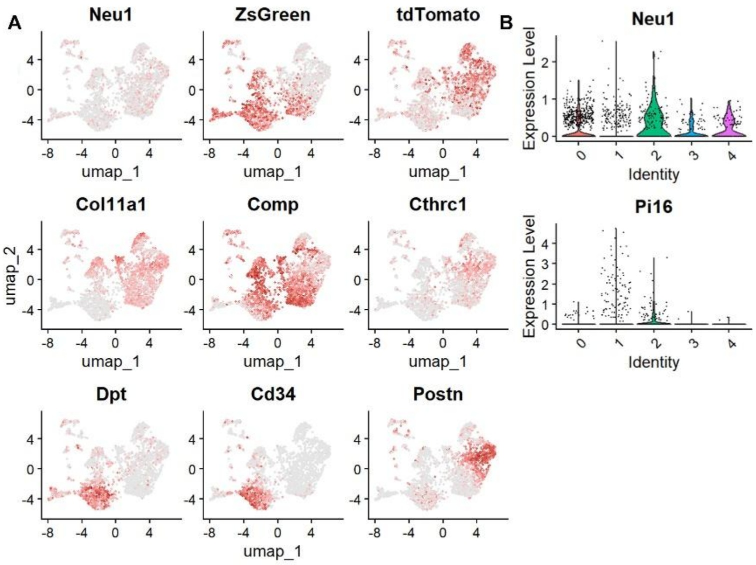


**Figure S9.** (A) Feature Plot shows the distribution characteristics of marker genes in various cell subpopulations. Among these, the original authors consider *ZsGreen+, Dpt+, Cd34+*, and *Pi16* as markers for the periosteal fibrous layer, while *tdTomato+* and *Postn+* are markers for the periosteal cambium layer. (B) Violin plots show that there is expression consistency between the high-expression subpopulations of *Neu1* and *Pi16* (the fibroblast marker gene identified by the original authors).

**Figure S10**

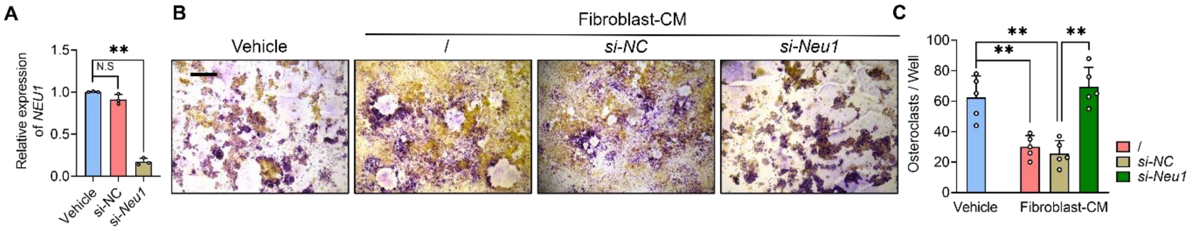


**Figure S10.** (A) Relative expression of *NEU1* mRNA in fibroblasts treated with vehicle, control siRNA (*si-NC*), or Neu1-targeting siRNA (*si-Neu1*), normalized to *Gapdh*, n = 3; (B) Representative TRAP staining images of osteoclasts cultured in conditioned medium produced by fibroblasts treated with vehicle, *si-NC*, or *si-Neu1*, scale bar = 200 um; (C) Quantification of osteoclasts per well in different treatment group, n = 5;

**Figure S11**

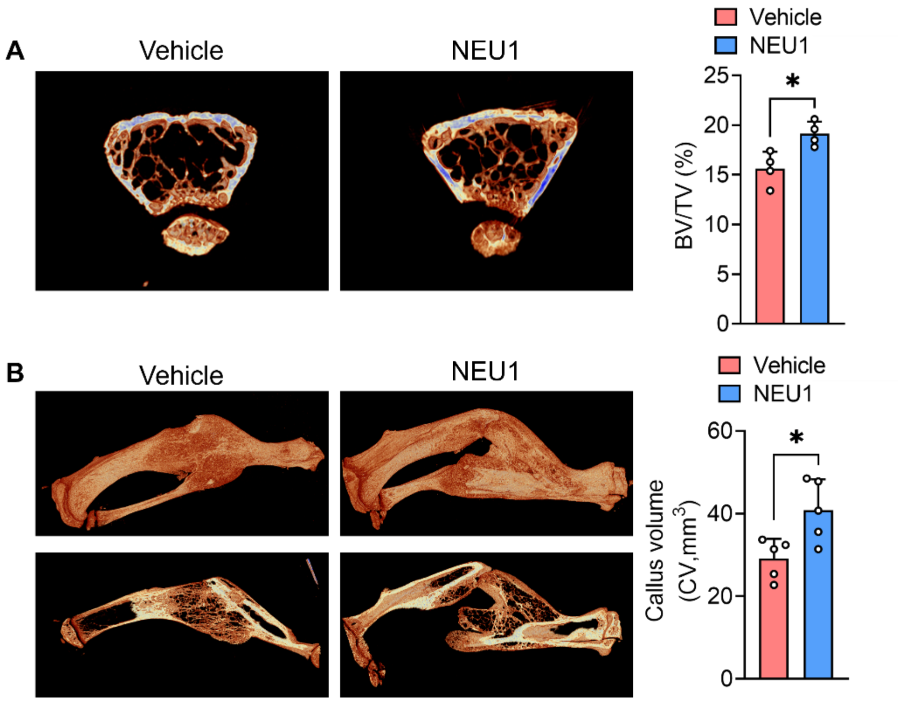


**Figure S11.** (A) Representative micro-CT images show cortical and trabecular bone of OVX model mice after treatment with vehicle or NEU1 homologous protein for 4 weeks; BV/TV (Bone bone volume fraction) analysis of trabeculae bone in both groups, n = 4. (B) Representative micro-CT images showing callus of fracture mice after treatment with vehicle or recombinant NEU1 for 4 weeks, quantification of CV (callus volume) from each group, n = 5;
